# Pigment Pathways Collide: A Rare Case of Retinitis Pigmentosa and Nevus of Ota

**DOI:** 10.7759/cureus.90369

**Published:** 2025-08-18

**Authors:** Divyashri R Nagarajan, Sathya J Kakade

**Affiliations:** 1 Ophthalmology, Burjeel Medical City, Abu Dhabi, ARE

**Keywords:** genetic eye disorders, nevus of ota, ocular melanosis, rare co-occurrence, retinitis pigmentosa

## Abstract

Nevus of Ota (ocular melanosis) is a dermal melanocytosis involving the ophthalmic and maxillary regions and can present with ocular involvement as melanosis oculi. Retinitis pigmentosa (RP) is a hereditary retinal dystrophy characterized by progressive photoreceptor degeneration. We report the first documented rare case of a 59-year-old female patient with a history of treated breast carcinoma who presented for an eye evaluation due to vision loss. Ocular examination revealed bilateral RP and unilateral ocular melanosis (Nevus of Ota). No established syndromic or genetic links between Nevus of Ota and RP exist. While pigmentation disorders such as albinism or syndromes like Waardenburg syndrome may involve both melanin pathways and retinal degeneration, Nevus of Ota has not been associated with retinal dystrophies in the literature. This case explores whether this co-occurrence may suggest a rare, undocumented syndromic association or is merely coincidental.

## Introduction

Nevus of Ota (oculodermal melanocytosis) is a benign condition characterized by bluish-gray hyperpigmentation of the periorbital skin and ocular structures due to dermal melanocyte proliferation. It may involve the sclera, conjunctiva, uvea, and periocular skin [1]. The first and second divisions of the trigeminal nerve, namely the ophthalmic and the maxillary, are most commonly involved. The Asian population is more commonly involved, affecting 0.014% to 0.034% of the population. Most commonly, they are present at birth, but lesions can also appear in puberty or during pregnancy due to hormonal changes. Females are more commonly affected than males at a ratio of 5:1 [2]. 

Retinitis pigmentosa (RP) represents a group of genetically heterogeneous retinal dystrophies characterized by progressive degeneration of rod and cone photoreceptors [3]. Symptoms include nyctalopia (night blindness), peripheral vision loss, and eventually central vision impairment. Its estimated prevalence worldwide is 1/4000. Diagnosis is based on characteristic fundus findings such as bone spicule pigmentation (clumps of dark pigment in the retina resembling spiky bone fragments), attenuated retinal vessels, and waxy optic disc pallor. Optical coherence tomography (OCT) shows thinning of outer retinal layers, particularly the ellipsoid zone (the part of the retina corresponding to the light-sensitive portion of photoreceptors) and the interdigitation zone (where photoreceptors interface with the retinal pigment epithelium, similar to interlocking fingers). Visual field testing reveals progressive peripheral loss, often forming a ring scotoma [4].

Although ocular melanosis increases the risk of ocular melanoma and glaucoma [1], no known associations with hereditary retinal degenerations have been reported. A comprehensive literature search in PubMed, Scopus, and Google Scholar from database inception to August 2025, using the terms (“Nevus of Ota” OR “oculodermal melanocytosis” OR “ocular melanocytosis”) AND (“retinitis pigmentosa” OR “rod-cone dystrophy”). Titles, abstracts, and full texts were reviewed without language restrictions to identify any prior reports describing both conditions in the same patient. To our knowledge, this is the first reported case of a patient presenting with both ocular melanosis (Nevus of Ota) and bilateral RP.

## Case presentation

A 59-year-old female patient presented for ophthalmic evaluation due to progressive vision loss over the past month. Her past medical history was significant for estrogen receptor-positive breast cancer treated with surgery and aromatase inhibitors (anastrozole, exemestane), cataract surgery in the right eye six years ago, hypothyroidism, hyperlipidemia, and prediabetes.

**Figure 1:**
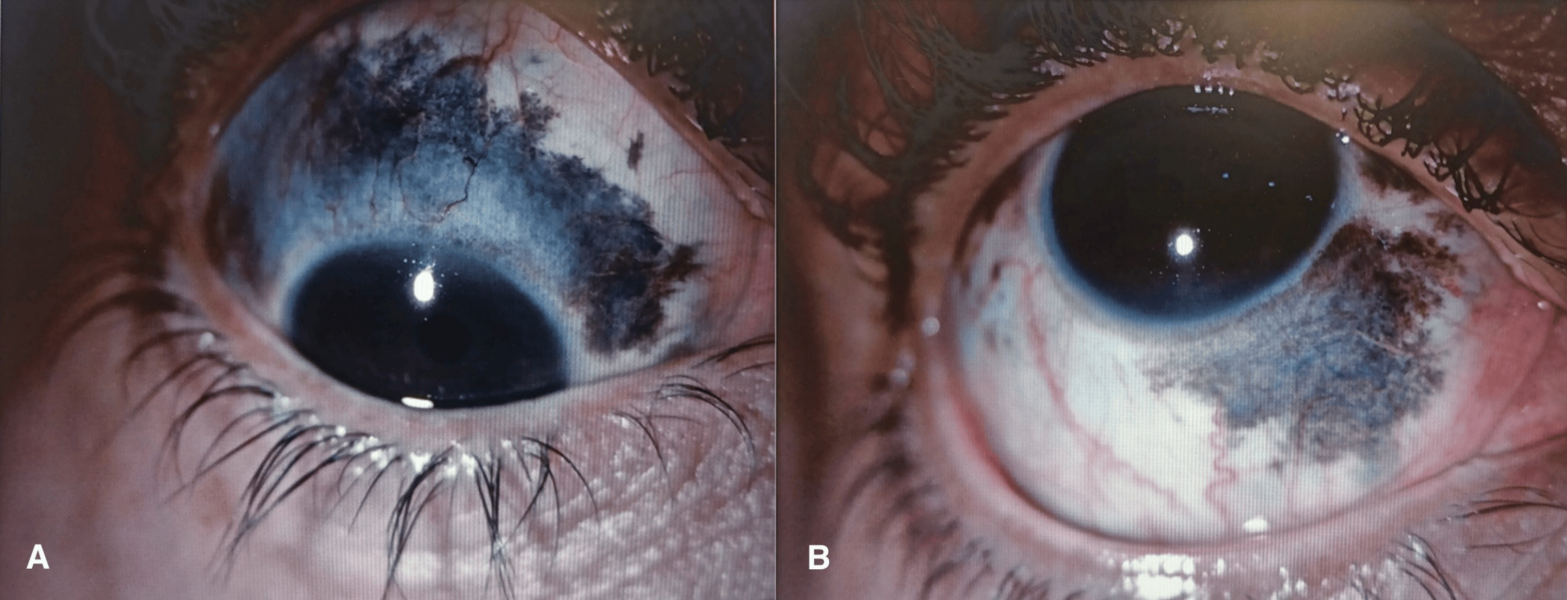
Anterior segment evaluation A: Conjunctival melanosis in the superior bulbar conjunctiva of the right eye; B: Conjunctival melanosis in the inferior bulbar conjunctiva of the right eye.

The patient underwent a comprehensive ocular examination. Visual acuity was reduced, measuring 20/100 in the right eye (OD) and 20/30 in the left eye (OS). Anterior segment evaluation (Figure 1) revealed conjunctival melanosis in the right eye (graded as melanosis++), a clear cornea and anterior chamber, and the presence of a posterior chamber intraocular lens (PCIOL) in the right eye, indicating prior cataract extraction. Pupils were round and reactive to light, and there was no relative afferent pupillary defect (RAPD). Intraocular pressure was within normal limits (13 mmHg OD, 11 mmHg OS). 

**Figure 2:**
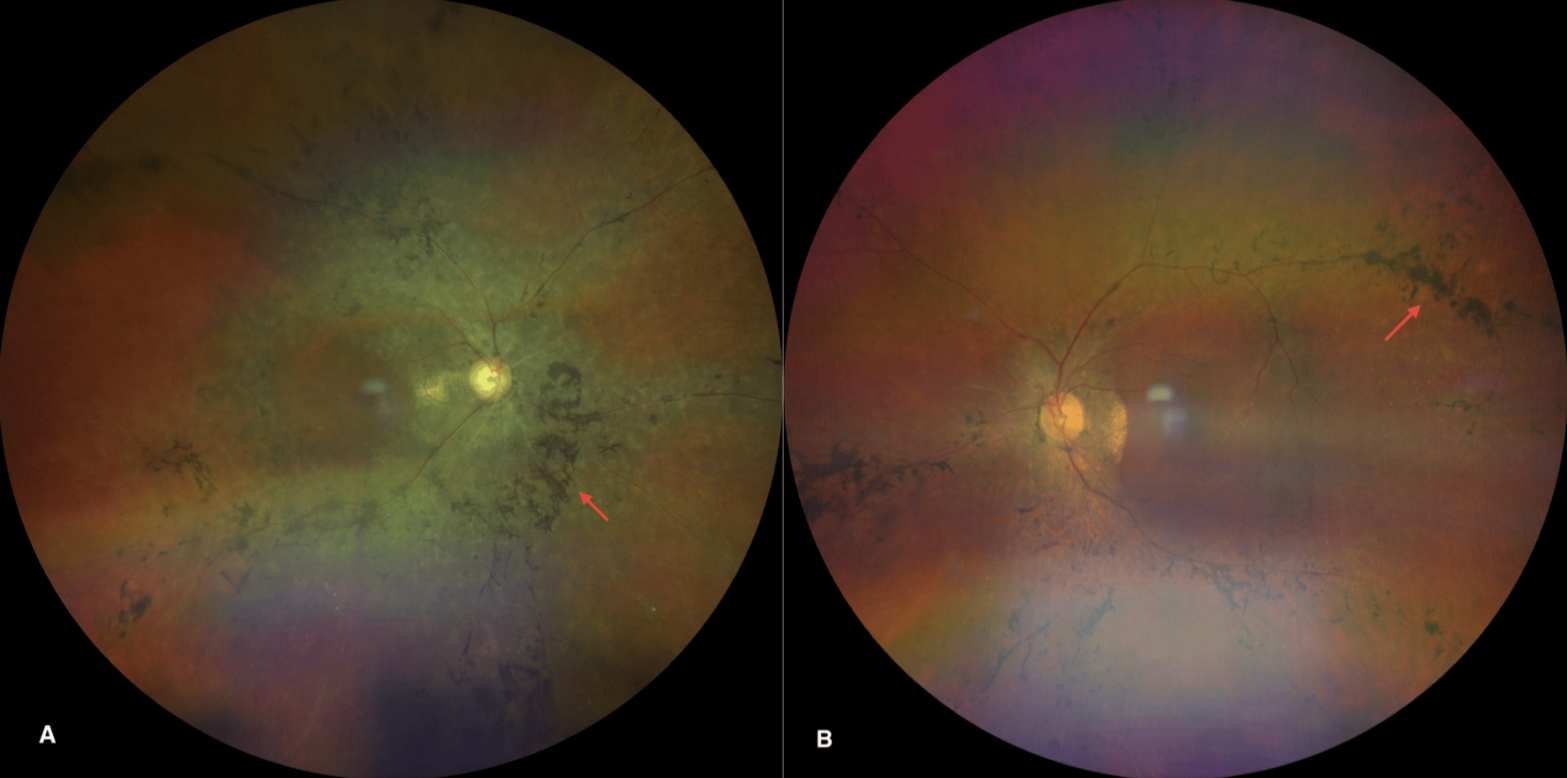
Fundoscopic examination showing characteristics of retinitis pigmentosa A: Bone-spicule pigmentation and vascular attenuation of the right eye; B: Bone-spicule pigmentation and vascular attenuation of the left eye.

**Figure 3:**
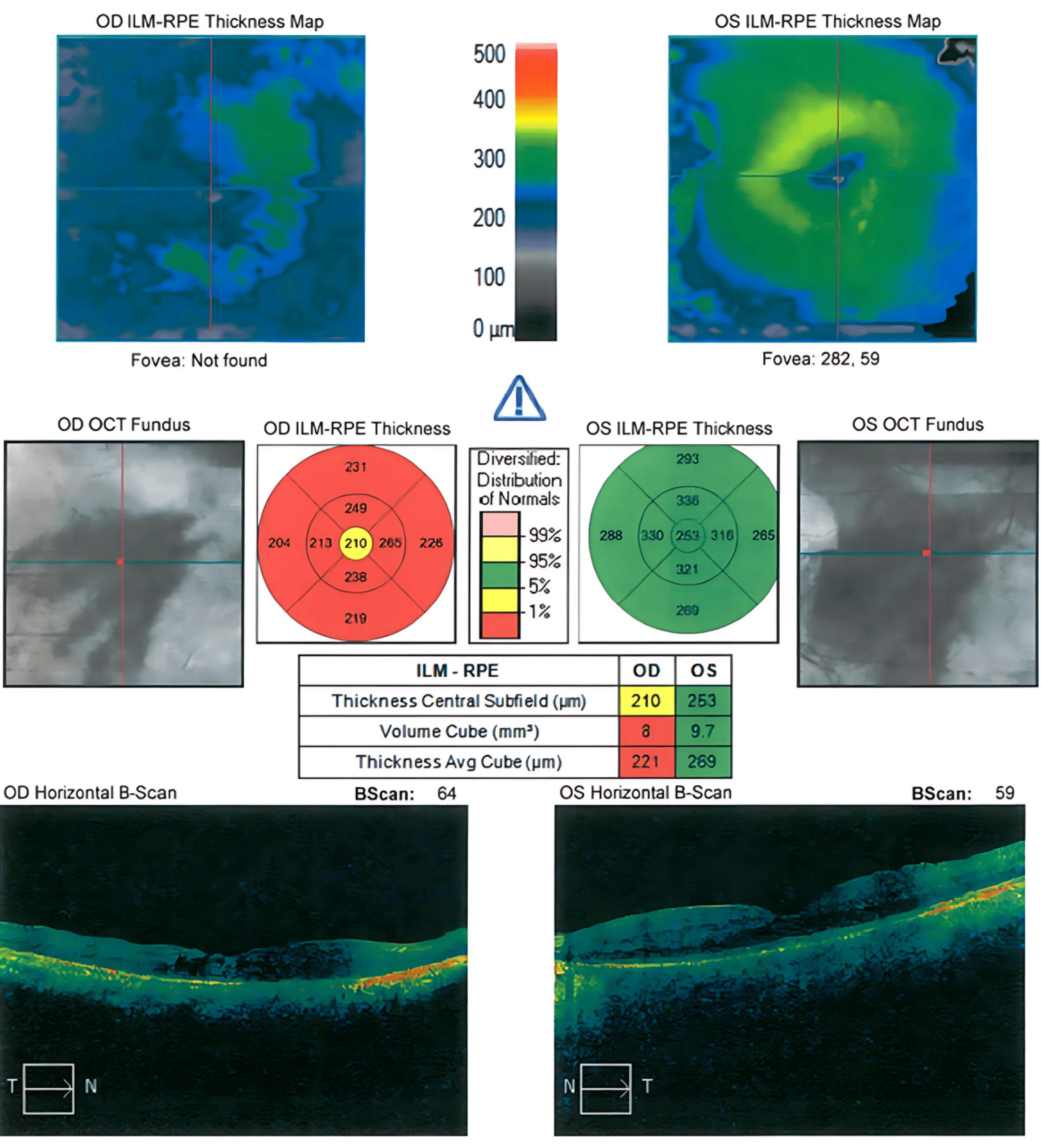
Optical coherence tomography (OCT) showing bilateral foveal atrophy, normal vitreoretinal interface, and no peripheral retinal degeneration OD: right eye: OS: left eye; ILM-RPE: internal limiting membrane-retinal pigment epithelium

Fundus examination showed classic features of retinitis pigmentosa in both eyes, including bone-spicule pigmentation and vascular attenuation, with preservation of the optic discs (Figure 2). OCT revealed foveal atrophy in both eyes, while the vitreoretinal interface appeared normal (Figure 3). There was no evidence of peripheral retinal degeneration. Based on these findings, the diagnoses of bilateral RP and right eye ocular melanosis (Nevus of Ota) were made.

She was advised to have visual rehabilitation with glasses and to continue dry eye management with sodium hyaluronate drops and periodic ophthalmologic monitoring. Genetic counseling was considered but not pursued due to patient preference and resource limitations.

## Discussion

While both Nevus of Ota and RP involve pigmentation-related structures, their pathogenesis appears unrelated. Nevus of Ota involves the proliferation of melanocytes in the dermis and sclera, without known retinal involvement [1]. RP involves genetic mutations affecting photoreceptor or retinal pigment epithelium (RPE) function [3]. Although RP usually progresses slowly over decades, our patient experienced a relatively rapid decline in vision, which may reflect late presentation after unnoticed progression, an atypically aggressive RP phenotype, or functional impact from coexisting ocular melanocytosis.

Syndromes such as Waardenburg [5], Albinism, and Usher syndrome may show overlapping pigmentary and retinal manifestations [6, 7], but Nevus of Ota has not been implicated in such syndromes.

Melanocyte-related genes such as microphthalmia-associated transcription factor (MITF) and SRY-related HMG-box gene 10 (SOX10) have been linked to pigmentation disorders with ocular manifestations [8]; however, RP is more commonly associated with the rhodopsin gene (RHO), usherin gene (USH2A), RP GTPase regulator gene (RPGR), and other retinal-specific genes [3]. No known shared genetic pathways currently link these conditions [5].

Given the patient's history of breast cancer and long-term use of aromatase inhibitors, one might also consider whether endocrine or paraneoplastic factors could have influenced retinal degeneration, though this remains speculative and undocumented.

The aim of reporting this case is to showcase a rare and possibly unprecedented co-occurrence of Nevus of Ota and RP in the same patient. To our knowledge, no definitive syndromic or genetic link between these two pigmentary disorders has been established in existing literature. By documenting this case, we hope to raise clinical awareness of such unusual presentations and prompt further research into potential shared developmental, genetic, or pigment-related pathways.

## Conclusions

Our case presents an unusual co-occurrence of ocular melanosis (Nevus of Ota) and RP, not previously documented in the literature. This report highlights the clinical importance of recognizing such rare presentations, as early identification may guide monitoring for potential complications and inform patient counseling. Whether this represents a coincidental finding or a syndromic overlap involving shared pathways of melanin biology and retinal degeneration warrants further investigation. In this context, comprehensive genetic testing could play a valuable role in elucidating underlying mechanisms, identifying potential hereditary risk factors, and contributing to personalized patient management. Case documentation and genetic testing in similar presentations may help establish or refute this possible association.
